# Baltimore Medical and Surgical Society

**Published:** 1881-12

**Authors:** J. H. Branham

**Affiliations:** Baltimore, Md.; Reporter for the Society


### Societies.

### BALTIMORE MEDICAL AND SURGICAL SOCIETY.

By J. H. BRANHAM, M.D., Baltimore, Md.
Reporter for the Society.

*For the Atlanta Medical Register.*

Dr. Rohe referred to several cases reported by Dr. A. B. Arnold at a previous meeting, which had been diagnosticated as *pernicious malarial fever*, and related a case which bore some similarity to the cases of Dr. Arnold.

The patient is a telegraph operator, about twenty-eight years old. He had been feeling well the day before, and had gone to work after eating a hearty dinner of omelet about four o'clock in the afternoon. About midnight, while at work, he was seized with nausea and a desire to go to stool. He went to the water-closet and had a free evacuation of the bowels. He was then seized with a fainting fit, during which he fell off the seat on to the floor. When he recovered he found that he had vomited and purged during his unconscious state. He returned to work, but in a short time was seized with an exactly similar attack. He then got in a carriage and came to Dr. Rohe's office, reaching there about half past one o'clock. On examination he was found in a state of collapse ; pulse rapid, weak and compressible ; skin covered with cold, clammy perspiration, and temperature 97.5° F. A hypodermic injection of one-third of a grain of morphia was given him, and in fifteen minutes his skin became warm and dry, and his pulse better. He was then taken home, and fifteen drops of deodorized tincture of opium in one drachm of aromatic spirit of ammonia ordered every two hours, with whisky *ad libitum.* In the morning he was found much improved, but very weak. Ordered thirty grains of quinine to be taken in the course of the day. His attack had not recurred, but he had been unable to return to work for nearly a week.

Dr. Rohe had been at first disposed to regard the attack as simply one of indigestion, but the violence of the symptoms and the absence of all pain, had suggested its similarity to the case reported by Dr. Arnold as pernicious malarial infection. There was no history in the case of a well-marked chill or fever.

Dr. Norris could see no connection between this case and malarial poisoning. He would call it a case of cholera-morbus.

Dr. Percival said in 1856 Alabama was devastated by a great hurricane, which blew a large amount of animal and vegetable matter into a lake near where he was practicing. During the next summer this began to decay, and at the same time a large number of the inhabitants of a neighboring plantation were attacked with a very fatal epidemic, which rapidly spread over a large section of country.

These attacks were characterized by a chill, followed by fever resembling ordinary malarial disease ; but on the next day, at the same time, instead of the recurring collapse, came on great thirst, contraction of the skin, rapid loss of flesh and a sensation of chilliness, accompanied by rapid pulse. The attacks which could be warded off by quinia in large doses, were never accompanied by vomiting or purging. They were called pernicious malarial fever.

Dr. Branham thought that we could always differentiate between cholera-morbus and malarial seizure, by the use of the thermometer, as in the one the temperature is always depressed, while in the other the internal temperature is always elevated.

Dr. J. S. Lynch said, "I have had lately under treatment a man aged twenty-eight, who was attacked on Sept. 6th with febrile symptoms, accompanied by redness of skin, which began on the neck and cheek, and rapidly extended over the entire body from the crown of the head to the soles of his feet. About the third day a great number of vesicles formed under the cuticle over the entire body. In a short time their contents became purulent, and finally the whole epidermis, including the nails, was shed. It came away in large flakes, so that a complete cast of the hands or feet could have been obtained.

"There was much bronchial irritation, and the urine was thick and dark. The patient was delirious, and the temperature was never under 102.5° F. I attributed this to the effects on the nervous system of the retained poisonous materials which should have been excreted by the skin.

"The treatment consisted of quinine, digitalis, and acetate of potassium. Under this the urine became abundant and clear and the delirium subsided. The exfoliation of the epidermis was completed in about ten days, and the patient was well, except that he had a slight bronchitis.

"On October 2d, a second attack occurred, which was similar to the first, but was more rapid in its course and milder. This time only part of the vesicles became purulent.

"The treatment consisted of veratrum viride, pushed to nausea.

"Two weeks later a still milder attack occurred, from which he recovered without treatment."

Dr. Rohe said that he had seen the case with Dr. Lynch, and while it presented a history differing in many particulars from any form of skin disease described in the books, he was disposed to regard it as a case of acute general eczema.

Dr. Lynch also reported a case of a man aged forty-seven, who had been treated for five months, and to within five weeks of his death, for dyspepsia. He saw him five weeks before his death, and immediately diagnosed cancer of the stomach.

He was assisted in making the post mortem by Dr. J. W. Chambers. It was made twenty-four hours after death. Part of the anterior wall of the stomach was destroyed so that there was an opening, one and a half by two inches in diameter, near the pylorus. The gall bladder was entirely destroyed and the edges of the opening in the stomach were adherent to the liver, so that none of its contents had escaped into the peritoneal cavity Two large gall stones were found in the stomach. Death, which had occurred suddenly, was caused by erosion of one of the gastric arteries.

Dr. Rohe stated that he had recently seen quite a large number of cases of *impetigo contagiosa*, which is ordinarily rather infrequent in this city. This diease which was first described by Dr. Tilbury Fox thirteen or fourteen years ago, was characterized by the formation of large vesicles and bullæ, the contents of which were either serous or sero-purulent. The bullæ are formed rather rapidly and undergo a rapid involution. In the course of a day—sometimes within a few hours—the greater portion of the fluid became resorbed and the bullæ presented a flaccid appearance. After completely drying up, a thin scaly crust remained, which turned up at the borders, and only adhered at the centre. In a few days these crusts drop off leaving a light brown pigmentation which gradually disappears.

If the eruption is not irritated by scratching or stimulant applications, the course described is followed by the affection, and no active treatment is required. Sometimes, however, in consequence of considerable itching which may be present, an eczema is set up by scratching, which demands appropriate remedies.

The cau<sup>s</sup>e of tne affection is obscure, but it is probably parasitic. It is contagious, and usually affects all the children of a family in succession. It can be inoculated and reproduced on a healthy person in several generations. Some years ago, Dr. Rohe had inoculated himself with the fluid from one of the bullæ in a dispensary patient, and had reproduced others by inoculating himself with the contents the bulla first developed on his body. He had examined the fluid and crusts carefully with the microscope, but had failed to find any spores or mycelial elements to indicate the parasitic nature of the disease. Several dermatologists of repute had cl imed that such evidence of the parasitic character of the disease existed, but it was generally doubted.

The diagnosis might be difficult when first seen, between a bullous syphilide or pemphigus. If the patient could, however, be kept a few days under observation these difficulties would disappear, and the diagnosis be cleared up. The eruption always disappears in two or three weeks unless irritated, or when it occurs in an anæmic, broken-down child, then it may terminate in a persistent pustular eczema.

The treatment was of the simplest description; an application of calomel ointment, or benzoated oxide of zinc ointment seemed to have some influence in hastening its disappearance; constitutional remedies of course, were not necessary unless the general condition of the patient called for them.

Dr. Brinton said, "I was consulted a few weeks since, by a Miss H., of German parentage for what she termed 'spots' on her lower extremities, abdomen and arms. They had appeared and disappeared several times during the last four or five months. As the trouble comes on at regular intervals of about twenty-eight days her mother thought that it was connected with her menses which had never appeared. She was well except that on the day of the appearance of the 'spots,' she suffered with headache, pain in the back, etc. She is decidedly anæmic.

"On examination I found thousands of the purpuric extravasations, which varied in size from a pin's head to a ten-cent silver piece.

"The spots disappeared in a few days undergoing the changes usual in extravasated blood. Just twenty-three days from the time she first consulted me, she had the same trouble again; and still another attack twenty-six days after this, each crop disappeared in one week. The etiology of purpura is very obscure, it being given as 'hepatic congestion,' 'internal obstruction,' 'enlarged spleen,' 'debility of the nervous powers,' 'deficient or suppressed menstruation,' by various authors.

"The only case similar to the above which I have read of is reported by Pifford, of New York, in his book, 'The Materia Medica and Therapeutics of the skin.'

"I cannot think that this regular occurrence of a crop of purpuric spots was due to chance. The only other explanation is that it is vicarious menstruation, and it is certainly as natural to say that when the menses are suppressed the blood may escape under the skin as to say it escapes from the nose.

"I still have the case under observation and will report further progress at some other meeting."

Dr. Rohe doubted the propriety of ascribing the eruption to vicarious menstruation. The occurrence of vicarious menstrual flow was not sufficiently well established to justify its acceptance as the cause of the eruption in the case narrated. Besides the interval between the first and second, and second and third eruption was not sufficiently regular to be regarded as typical of a recurrent menstrual period. The eruption might have been feigned, that is, designedly produced, or its reappearance at the times noticed might have been a pure coincidence.

In the discussion which followed the reading of Dr. Chambers' paper on Tracheotomy in Diphtheria (See page 129), Dr. Caldwell remarked that he thought the operation was generally done too late, but he would always advise it to mitigate the sufferings of the little patient if for nothing else.

Dr. Morfit asked if the membrane had extended below the opening in many of the cases.

Dr. Chambers—" It had in all."

Dr. Morfit—"I do not think that local treatment can cure diphtheria, which is a blood disease. The tube acts as an irritant, and the operation is apt to cause capillary bronchitis and prove fatal in consequence." He considers the cases where the larynx is invaded as almost certainly fatal, and thinks the reported recoveries are instances of mistaken diagnosis.

Dr. Scarff—" I think that the purpose of the operation is to gain time so that we may cure the disease by internal medicines, and is not in itself supposed to be curative.

Dr. Rohe thought that we had learned something about the clinical history of diphtheria. If anything about it was proven it was that laryngeal diphtheria was in most cases fatal.

Now, the statistics of Dr. Chambers and of Drs. Fenger and Lee, of Chicago, and others, lately published, show that about one-third of the cases operated on by tracheotomy are saved.

He (Dr. R.) would like to ask Dr. Morfit whether it was not worth while to try to save one case out of three of laryngeal diphtheria?

It was unfair to charge tracheotomy with causing bronchitis and pneumonia, because those diseases occurred in cases in which the operation was not performed.

Dr. Branham thought that common sense and physiological reasoning are often better guides when considering the propriety of a surgical operation than statistics, which are necessarily imperfect. In this disease, where the larynx is partly closed and an insufficient amount of air gets to the lungs, we have, in addition to the blood-poisoning, a far more rapid and certain cause of death—the want of oxygen in the blood. Again, the powerful efforts which the child makes to breathe, with the impossibility of the entrance of air, produces more or less of a vacuum in the thorax, and in consequence, a large amount of blood accumulates there. This, if it is continued, will lead to ordinary bronchitis or pneumonia, and rapidly prove fatal. Now, by an operation, which is confessedly not dangerous in itself, we can ward off or arrest these perils.

In view of these facts, he was surprised that any one should object to tracheotomy, and could but believe that the profession will soon be unanimous in recommending its employment under such circumstances.

